# Equine adipose-derived stem cells modulate *in vitro* neutrophil extracellular trap release by polymorphonuclear neutrophils

**DOI:** 10.3389/fvets.2025.1685757

**Published:** 2025-10-22

**Authors:** Constanza Salinas-Varas, Gabriel Espinosa, Tamara Muñoz-Caro, Iván Conejeros, Ulrich Gärtner, Kerstin Fey, Stefan Arnhold, Anja Taubert, Carlos Hermosilla

**Affiliations:** ^1^Institute of Parasitology, Faculty of Veterinary Medicine, Justus Liebig University Giessen, Giessen, Germany; ^2^Escuela de Medicina Veterinaria, Facultad de Medicina Veterinaria y Recursos Naturales, Universidad Santo Tomás, Talca, Chile; ^3^Institute of Anatomy and Cell Biology, Justus Liebig University Giessen, Giessen, Germany; ^4^Equine Clinic, Internal Medicine, Justus Liebig University Giessen, Giessen, Germany; ^5^Institute of Veterinary Anatomy, Histology and Embryology, Faculty of Veterinary Medicine, Justus Liebig University Giessen, Giessen, Germany

**Keywords:** MSCs, ADSCs, neutrophils, NET, horse

## Abstract

Neutrophil extracellular trap (NET) are thin and long web-like structures composed of DNA and antimicrobial proteins released by activated polymorphonuclear neutrophils (PMN) as part of the innate immune response. Adipose-derived stem cells (ADSCs) represent an accessible, abundant and minimal invasive source of mesenchymal stem cells (MSCs), with high regenerative potential, immunomodulatory and anti-inflammatory properties. Although recognized immunomodulatory properties of ADSCs, their interaction with PMN and their role on NET formation remains poorly characterized. The present study aimed to evaluate the *in vitro* effects of equine ADSCs on NET formation by equine PMN. Equine ADSCs were isolated from two different sources of adipose tissue, subcutaneous and retroperitoneal adipose stores. Equine PMN were isolated from peripheral blood with a discontinuous density gradient and stimulated with phorbol 12-myristate 13-acetate (PMA) to induce NET release as positive control. Scanning electron microscopy (SEM) and immunofluorescence microscopy (IFM) analyses were performed to assess NET release by equine PMN co-cultured with ADSCs. *In vitro* IFM-NET quantification revealed a significant NET decrease for PMN co-cultured with ADSCs and PMA. Furthermore, extracellular DNA quantification showed that inhibition of equine NET is dependent on the ADSCs to PMN ratio, for PMA and ionomycin stimulated PMN. Moreover, our findings unveil no modulation of reactive oxygen species (ROS) production by equine PMN when co-cultured with ADSCs. In summary, our results provide evidence of ADSCs on equine PMN, particularly in their capacity to attenuate NET formation and release. These results support the potential role of ADSCs on host innate immune response and thereby maintaining immune homeostasis. Further investigation is needed to better understand the specific molecular pathways involved in NETosis via ADSCs.

## 1 Introduction

Mesenchymal stem cells (MSCs) are multipotent stromal cells characterized by their capacity for self-renewal and differentiation into a broad range of cell types, including osteocytes, chondrocytes, and adipocytes (1). Although MSCs were firstly isolated from bone marrow samples, it is well known that these highly plastic adherent cells can be obtained from diverse sources, including the umbilical cord, placenta, peripheral blood and adipose tissue (2). Adipose-derived stem cells (ADSCs), a subset of MSCs, have gained significant attention in veterinary medicine due to their low immunogenicity and high accessibility with minimal ethical concerns (3). As such, ADSCs represent an accessible, abundant and minimal invasive source of MSCs, with high regenerative potential as well as immunomodulatory and anti-inflammatory properties (4). These properties have been linked to a variety of MSC-derived bioactive molecules, such as growth factors, cytokines, chemokines and extracellular vesicles (5). The strong immunomodulatory properties of MSCs, mainly driven through paracrine signalling, have been associated with the secretion of key molecules such as prostaglandin E2 (PGE2), transforming growth factor-beta (TGF-β) and interleukin-10 (IL-10) (6). Therapeutically, MSCs have shown promising results in humans promoting tissue regeneration and wound healing, enhancing fibroblast migration, and treating degenerative and inflammatory disorders such as osteoarthritis and colitis (7–9). In animals, ADSCs have shown therapeutic promise in musculoskeletal disorders, wound healing, and immunopathologies across multiple species, including dogs, cats, horses and cattle (4, 10). In equine medicine, autologous and allogeneic ADSCs are under active clinical investigation with high impact on musculoskeletal and orthopaedic injuries such as suspensory ligament desmitis, superficial digital flexor tendonitis, and degenerative joint diseases such as osteoarthritis (11–13). Equine ADSCs share phenotypic and functional characteristics with their human counterparts, expressing classical MSC surface markers such as CD90+, CD73+, and CD105+, with the unique expression of CD146+ and CD34+ (14–16). Functionally, MSCs exert a wide range of immune-modulatory properties in both, the adaptive- and the innate immune system, enhancing cell shift from pro-inflammatory to anti-inflammatory profiles in monocytes and macrophages, supressing B and T lymphocyte proliferation with inhibition of natural killer cells (NKs) among others (17, 18). Despite promising data on MSCs therapies, the mechanisms underlying their immunomodulatory effects, particularly in the modulation of innate immune cells like polymorphonuclear neutrophils (PMN), remains poorly explored (6, 19).

PMN are among the most abundant and first white blood cells of mammals to be recruited to sites of infection and/or injury (20). PMN exhibit diverse and essential defense mechanisms, which include phagocytosis, degranulation, production of reactive oxygen species (ROS) and the release of neutrophil extracellular trap (NET) (21). NETs are thin and long web-like structures composed of nuclear and mitochondrial DNA, histones and granule proteins such as myeloperoxidase (MPO) and neutrophil elastase (NE), released to entrap and neutralize pathogens (22). However, excessive or dysregulated NET release can significantly contribute to the pathogenesis of autoimmune and infectious diseases (23, 24), conditions in which MSCs may play a key role helping promote their resolution (25, 26). Despite growing evidence of MSC-derived immunomodulatory properties, their direct interaction with PMN and influence on NET formation and release remains poorly characterized. So far, research had mainly focused on other PMN defense mechanisms such as chemotaxis, ROS production, phagocytosis and PMN lifespan (27–29). *In vitro* studies in humans have demonstrated supportive effect of MSC-derived molecules on PMN lifespan and oxidative burst response (29, 30). Divergent findings have been reported regarding the impact of MSCs on the phagocytic capacity of human PMN (29, 31). Regarding NETosis, an *in vitro* reduction in NET released by human PMN in the presence of MSCs has been reported (31, 32). In horses, an increased PMN viability (33), decreased ROS production with no changes on PMN phagocytic capacity or NET production when exposed to bone marrow-derived MSCs has been described (34). The interplay between MSCs and host innate immune system, specifically on PMN regarding NET formation remains poorly understood. The limited data available highlights the need for further *in vitro* and *in vivo* studies to optimize MSCs clinical applications in veterinary medicine. Thus, the aim of the present study was to evaluate the *in vitro* immunomodulatory effect of equine ADSCs on NET formation by exposed equine PMN. Hereby, we seek to contribute to a more comprehensive understanding of ADSCs interactions with PMN as well as PMN-derived NET formation in horses.

## 2 Material and methods

### 2.1 Ethics statement

All animal procedures were performed according to the Justus Liebig University Giessen Animal Care Committee guidelines, approved by the Ethic Commission for Experimental Animal Studies of the State of Hesse (Regierungspräsidium Giessen) and in accordance to the current German Animal Protection Laws.

### 2.2 Blood samples and equine PMN isolation

Peripheral residual blood samples were obtained from clinically healthy adult horses (*n* = 3) by jugular venipuncture and processed within 1 h after sample collection. For equine PMN isolation, a discontinuous density gradient (Percoll™ GE Healthcare #17089101, Cytiva) was performed as previously described by Siemsen et al. (35). Briefly, 85% density Percoll and 70% density Percoll were layered in a 15 mL tube (#188271-N, Greiner, Germany), followed by 4 mL of whole blood on top. Gradients were centrifuged at 450 × g with no brake at room temperature (RT) for 45 min. After this time, the band containing the PMN in the interface between the two gradients was collected and washed thrice with sterile phosphate-buffered saline (PBS) solution. Cells were centrifugated at 450 × g with no brake at RT for 10 min. Finally, cells were gently suspended in a modified Roswell Park Memorial Institute (RPMI 1640) medium without phenol red (#R7509, Sigma-Aldrich) and counted with a Neubauer haemocytometer chamber (Blaubrand, Germany). Cells were maintained on ice until further use.

### 2.3 Isolation and cultivation of equine adipose-derived stem cells (ADSCs)

Adipose tissue was harvested from the region above the dorsal gluteal muscle (subcutaneous adipose tissue) as previously described by Raabe et al. (36), and from the retroperitoneal space from mixed breed male and female horses (aged mean +/− 4.74). While subcutaneous fat samples were obtained from horses slaughtered at the local abattoir, all other adipose tissue samples were obtained from horses undergoing abdominal surgery at the Equine Clinic, Department of Surgery at the Faculty of Veterinary Medicine, Justus Liebig University of Giessen, Germany. After harvested, adipose tissue of the different collection sites was diced into small pieces and washed in an equal volume of sterile PBS supplemented with 1% penicillin/streptomycin (Sigma-Aldrich). For cell isolation the adipose tissue was minced and then underwent enzymatic digestions using collagenase type I (Sigma-Aldrich) dissolved in BSA (bovine serum albumin; Sigma-Aldrich). The adipose tissue within the enzymatic solution was placed in a water bath at 37 °C for 10 min and afterwards in an incubator for about 30 min. The digested adipose tissue was centrifuged for 5 min at 260 × g. Afterwards the pellet was squashed through a 70 μm nylon cell strainer mesh (VWR International) and then pipetted into a 50 mL Falcon tube. The cells were washed in sterile PBS at 300 × g for 5 min and suspended in 1.0 mL of Dulbecco’s Modified Eagle Medium (DMEM; Gibco™) and 10% fetal calf serum (FCS; Gibco™). Finally, collected cells were transferred to a culture dish at a density of 250.000 cells per cm2. After 24 h, cell cultures were washed with PBS to remove non-adherent cells. Equine ADSCs used in this study were thoroughly examined for the expression of relevant stem cell markers such as CD90 and CD105 (14–16, 56). When cell cultures reached 80% of confluence, cells were detached from culture plates using TrypLE Express Enzyme® (Thermo Fisher Scientific), washed with DMEM (Sigma-Aldrich) and seeded at the desired amount in new plates to proceed with the experiments. Exclusively ADSCs obtained from retroperitoneal stores were used in experiment settings as these cells showed a better three MSC-lineage, i. e. adipogenic-, osteogenic- and chondrogenic differentiation capacities.

### 2.4 Scanning electron microscopy (SEM)

Isolated equine PMN (*n* = 3; 2 × 10^5^) were co-cultured with equine ADSCs (2 × 10^4^) on poly-L-lysine (0,001%, #P8920, Sigma-Aldrich) pre-coated coverslips for 3 h at 37 °C in presence or absence of phorbol-12-myristat-13-acetat (PMA; final concentration 2 μM; #P1585, Sigma-Aldrich). After incubation, cells were fixed in 2.5% glutaraldehyde (60 min, RT, Merck), post-fixed in 1% osmium tetroxide (Merck), washed in distilled water, dehydrated, critical point dried by CO_2_-treatment and spayed with gold. All samples were examined with a Philips XL30 scanning electron microscope at the Institute of Anatomy and Cell Biology at the Faculty of Human Medicine, Justus Liebig University Giessen, Germany.

### 2.5 Immunofluorescence microscopy (IFM) analysis of equine PMN-NET formation modulate by ADSCs

Unstimulated equine PMN (5 × 10^5^; *n* = 3) were co-incubated with ADSCs (5 × 10^4^) in a 10:1 ratio to analyze ADSCs-NET modulation. Equine PMN were seeded in a 24-well plate (#353047, Greiner) with pre-coated 10 mm coverslips (0,001% poly-L-lysine, #P8920, Sigma-Aldrich) and incubated with equine ADSCs (5 × 10^4^) at 37 °C for 15 min prior to addition of PMA (2 μM; #P1585, Sigma-Aldrich). After 3 h of co-culture, cells were fixed with 4% (w/v) paraformaldehyde (# 4979.1, Roth) at RT for 15 min. Then, samples were washed thrice with sterile PBS (pH 7.4) and incubated in blocking/permeabilization buffer (PBS; 3% BSA; 0.3%, Triton X-100; all Sigma-Aldrich) for 1 h at RT. Samples were incubated overnight at 4 °C with classical NET markers, including the primary antibodies anti-NE polyclonal antibody (1:300, rabbit, #Ab68672, Abcam) and anti-histone clone H11-4 monoclonal antibody (1:300; mouse, #MAB3422, Millipore). Next day, coverslips were gently washed with sterile PBS (pH 7.4) and incubated for 30 min at RT protected from light with the secondary antibodies Alexa fluor 488 goat anti-rabbit IgG (1:500; A11008, Invitrogen) and Alexa fluor 594 goat anti-mouse IgG (1:500; A11005, Invitrogen). Finally, samples were mounted with antifading DAPI-Fluoromount-G® medium (#0100–20, Southern Biotech). Images were acquired using the Keyence compact fluorescence microscope (BZ-X series; Keyence Corporation) and the ReScan Confocal instrumentation (RCM 1.1 Visible, Confocal.nl) combined with a Nikon Eclipse Ti2-A inverted microscope. Images were acquired applying identical brightness and contrast conditions and further processed with Image J Fiji version software (ImageJ 1.54f, USA). The percentage of NET-forming cells was quantified using the Hybrid Cell Count module of the Keyence BZ-X series fluorescence microscope. Events positive for pan-histone signal were divided by the total number of DAPI-positive cells and multiplied by 100 to calculate the percentage of NET-forming cells.

### 2.6 Total ROS production

Total ROS production was determined using the luminol-dependent chemiluminescence method (#A4685, Sigma-Aldrich). Equine PMN (*n* = 3; 1 × 10^5^) were suspended in RPMI 1640 medium without phenol red (#R7509, Sigma-Aldrich) and seeded in a 96-well sterile white flat bottom plate (#655074, Greiner). PMN were incubated with equine ADSCs (1 × 10^4^) at 37 °C for 15 min prior to the addition of the stimulus. Basal luminescence measurements were recorded after the addition of luminol (final concentration 7.5 μM), followed by the stimulation of the cells. In order to stimulate the release of NETs by equine PMN, neutrophils in the presence or absence of ADSCs were stimulated with PMA (2 μM; #P1585, Sigma-Aldrich). After stimulation, relative chemiluminescence units (RLU) were measured for 10 h. Experiments were performed in duplicates and chemiluminescence was measured via a luminometer (Luminoskan, Thermo Scientific).

### 2.7 NETs quantification on PMA/ionomycin-stimulated equine PMN in presence of different ratio of equine adipose-derived stem cells (ADSCs)

For the quantification of NET formation (*n* = 3) 1 × 10^5^ equine PMN per well were cultured alone (negative control) or co-cultured with equine ADSCs in a 96-well flat-bottom plate (Corning Life Sciences, Germany) at PMN: equine ADSCs ratios of 1,000:1, 100:1 and 10:1. Thereafter, equine PMN were treated with PMA (2 μM; #P1585, Sigma-Aldrich) or ionomycin (Io; 6 μM; #10634, Merck) for 3 h at 37 °C (positive control). After incubation, Sytox Orange nucleic acid stain (S-11368, Invitrogen) was added at a final concentration of 0.25 μM and plates were incubated for 5 min at RT. Culture medium containing Sytox Orange served as background fluorescence control. Following centrifugation at 400 × g for 5 min, half volume of supernatants were removed from each well and fluorescence intensity was measured immediately with an automated fluorometric reader (Varioskan Flash; Thermo Scientific) at excitation and emission wavelengths of 540 nm and 580 nm, respectively.

### 2.8 Statistical analysis

Statistical analyses were performed using the GraphPad Prism® software (GraphPad Software Inc., V10.4.1; La Jolla, CA, USA). Area under the curve (AUC) values were obtained for oxidative burst data. Normality of the data was assessed via the Shapiro–Wilk assay. *p*-values were determined by ordinary one-way ANOVA, followed by Tukey’s multiple comparison test. Data are presented as a mean ± standard deviation (SD). Statistical significance was defined at a *p* ≤ 0.05.

## 3 Results

### 3.1 ADSCs modulates NET release in equine PMN

**Figure 1:**
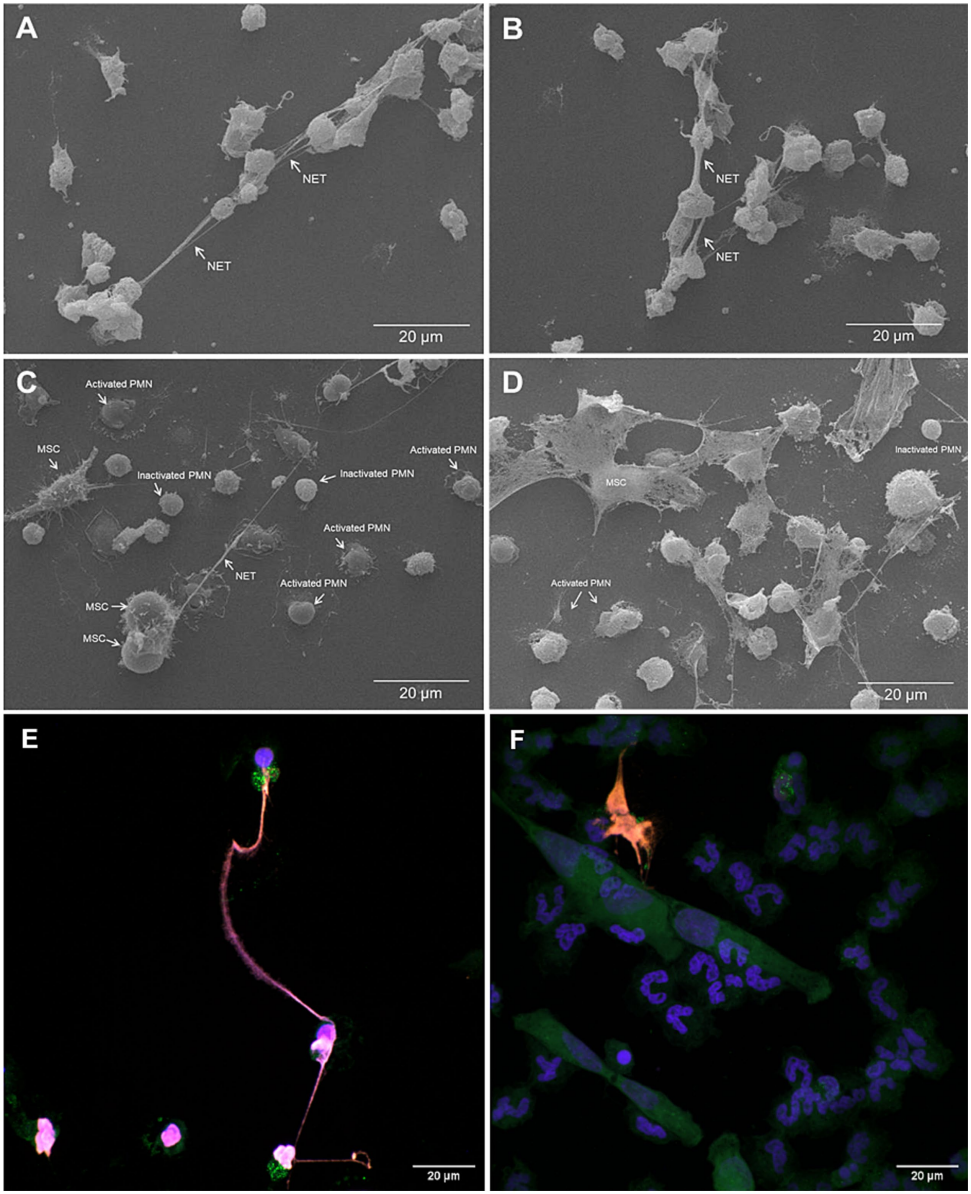
Scanning electron microscopy (SEM)- and immunofluorescence microscopy (IFM) analyses unveiled release of NET-like structures from equine polymorphonuclear neutrophils (PMN) exposed either to equine adipose-derived stem cells (ADSCs) and/or PMA. **(A,B)** SEM analysis revealed long and thin DNA strands consistent with NET-like structures released by equine PMN when stimulated by PMA for 3 h. **(C,D)** SEM images of PMA-activated PMN co-cultured with equine ADSCs for 3 h. **(E)** Confocal images of PMA-stimulated equine PMN in the absence or **(F)** presence of ADSCs. NET structures are visualized by co-localization of decondensed DNA (DAPI, blue), neutrophil elastase (NE, green) and pan-histone (red). Scale bar = 20 μm. MSC, mesenchymal stem cell; BF, brightfield microscopy.

**Figure 2:**
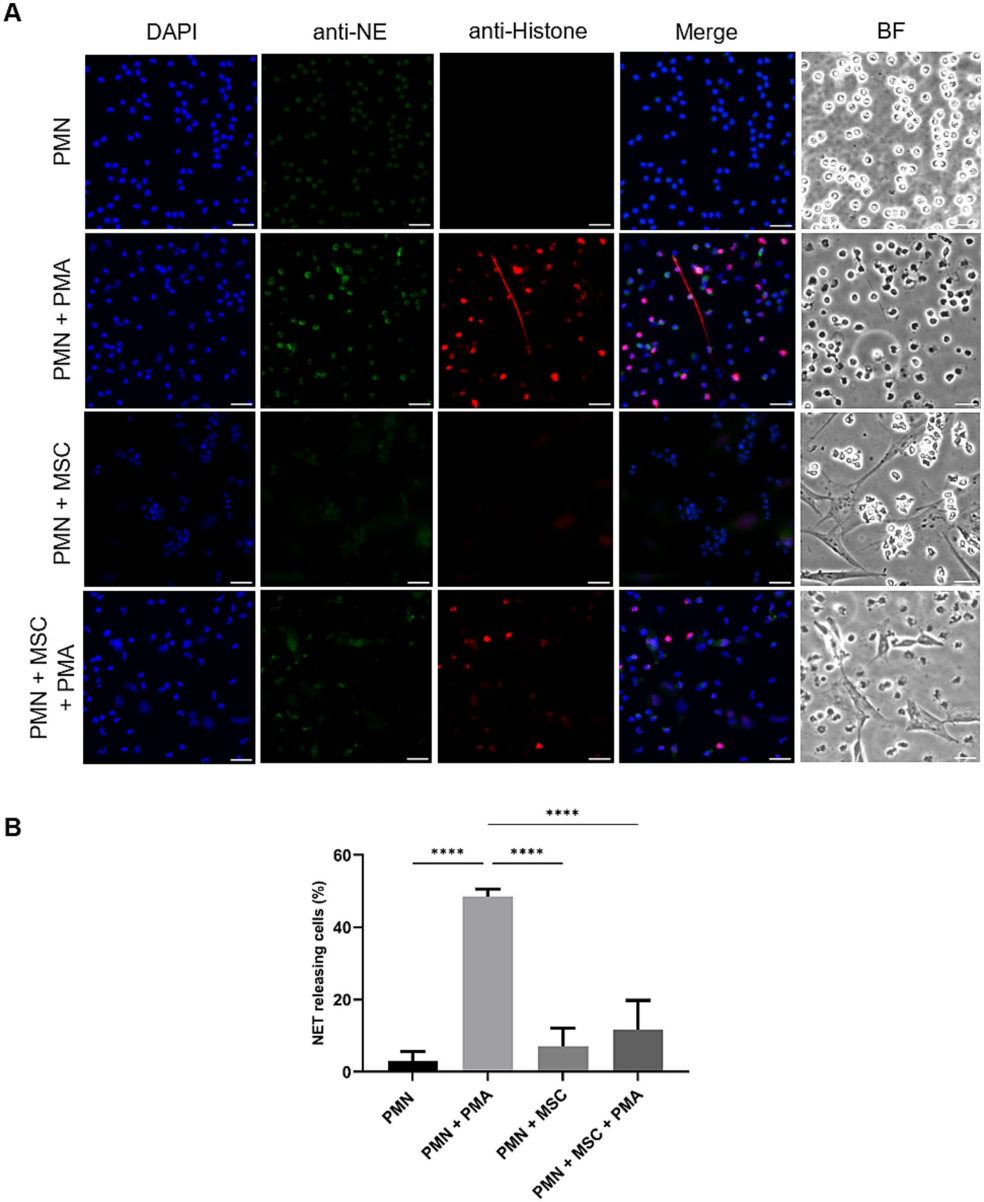
PMA-induced NET release by equine polymorphonuclear neutrophils (PMN) decreases in the presence of adipose-derived stem cells (ADSCs). PMN were cultured without stimuli (negative control), activated with PMA (positive control), in the presence or absence of equine-MSC for 180 min. **(A)** Immunofluorescence microscopy assay (IFM) analyses revealed NET structures by co-localization of decondensated DNA (DAPI, blue), neutrophil elastase (NE, green) and pan-histone (red). **(B)** The bar graph shows quantification of equine PMN releasing NETs. A significant difference (*p* < 0.05) was observed between PMA-stimulated PMN and those co-incubated with equine ADSCs and PMA. *p*-values were calculated by one-way ANOVA, followed by Tukey’s multiple comparison test: ****p ≤ 0.0001, ns, not significant. Scale bar = 20 μm. MSC, mesenchymal stem cell.

SEM- and IFM analyses revealed that ADSCs were capable of triggering equine PMN release of NET-like structures (Figure 1). To confirm functional immuno-modulatory capacities of ADSCs on equine NETosis, an IFM was performed. Co-localization of classical markers for NETosis, including extracellular DNA decorated with pan-histone and NE, confirmed the presence of equine NETs in the samples (Figure 2A). IFM quantification analysis revealed that PMA-stimulated PMN co-cultured in the presence of ADSCs resulted in no significant differences in the percentage of NET-releasing cells when compared to unstimulated equine PMN (*p* = 0.245), as shown in Figure 2B. On the other hand, a significant difference was observed between PMA-activated PMN (positive control) when compared to PMA-activated PMN co-cultured with ADSCs (*p* < 0.0001) (Figure 2B). Furthermore, no significant differences (*p* = 0.699) were observed between PMN co-cultured with ADSCs when compared to PMA-activated PMN co-cultured with ADSCs, confirming the anti-inflammatory modulatory effect of ADSCs on equine NETosis.

### 3.2 Equine ADSCs do not impair PMA-induced ROS production in equine PMN

**Figure 3:**
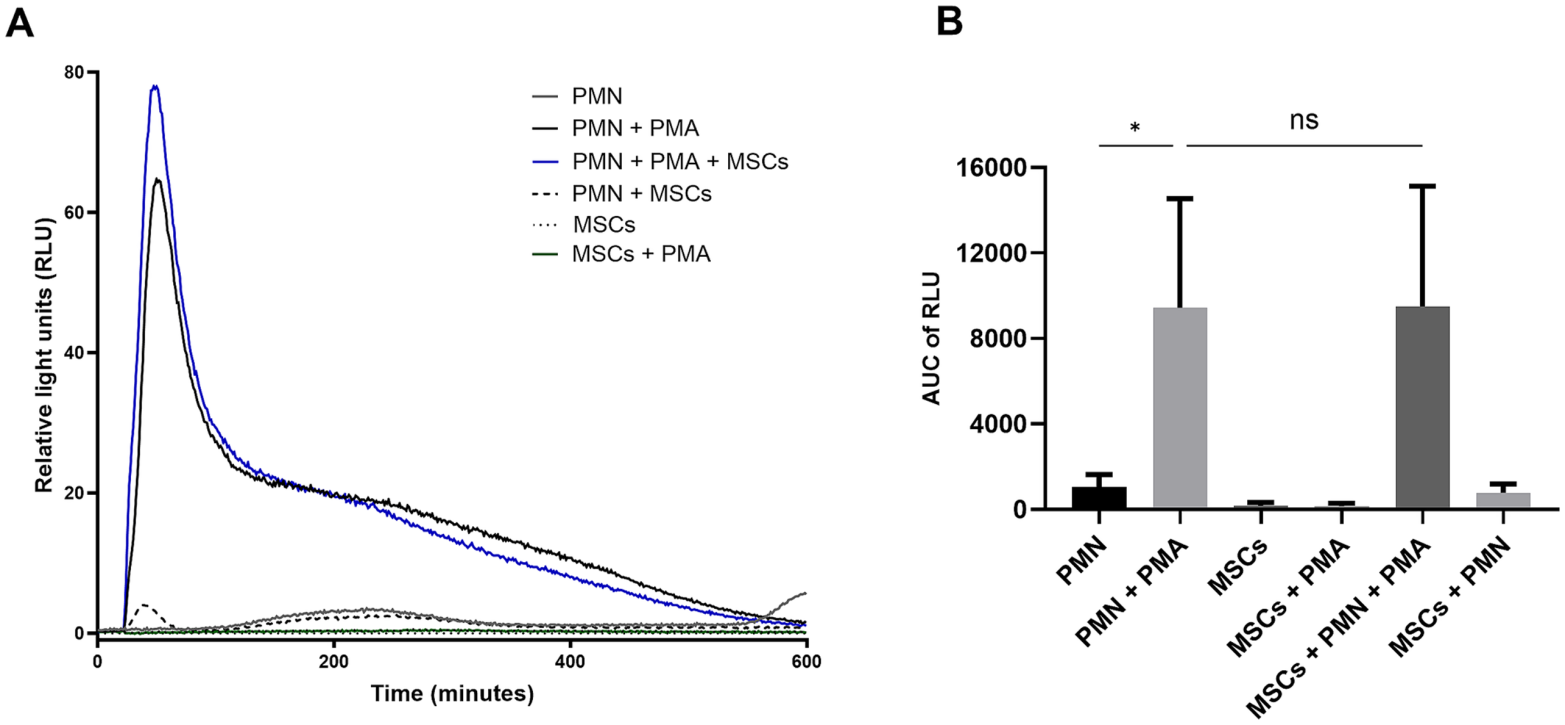
Equine adipose-derived stem cells (ADSCs) fail to modulate PMA-induced reactive oxygen species (ROS) production in equine polymorphonuclear neutrophils (PMN). PMN were co-cultured with ADSCs (10:1 ratio) and stimulated with PMA for 600 min. **(A)** Representative chemiluminescence registries illustrating ROS production in equine PMN. **(B)** Bar graph showing the area under the curve (AUC) of total ROS production. No significant differences (*p* > 0.05) were observed in ROS production by PMA-activated PMN when co-incubated with ADSCs, compared to PMA-activated PMN. Statistics analysis performed by one-way ANOVA, followed by Tukey’s multiple comparison test: *p ≤ 0.05, ns, not significant. MSC, mesenchymal stem cell.

To study the *in vitro* capacity of equine ADSCs to modulate PMN-driven ROS production, a luminol-based chemiluminescence assay was performed (Figure 3A). ROS production, after a 15 min co-culture incubation of equine PMN with ADSCs, was assessed. AUC values analysis showed a significant difference (*p* = 0.024) between unstimulated PMN (AUC: 1043 ± 583.9) and PMA-activated PMN (AUC: 9435 ± 5,104) (Figure 3B). No significant differences (*p* > 0.05) were observed between PMA-exposed PMN (AUC: 9435 ± 5,104) and PMA-activated PMN co-cultured in the presence ADSCs (AUC: 9505 ± 5,625) (Figure 3B). As a control, the AUC of unstimulated PMN co-cultured with ADSCs (AUC: 773.5 ± 425.2) was calculated, as MSCs are described to produce and release ROS under stress conditions (37) (Figure 3B). No significant differences (*p* > 0.05) were observed when this condition was compared with the unstimulated control PMN (AUC: 1043 ± 583.9) (Figure 3B).

### 3.3 Inhibition of equine NET release is dependent on the ADSCs to PMN ratio

**Figure 4:**
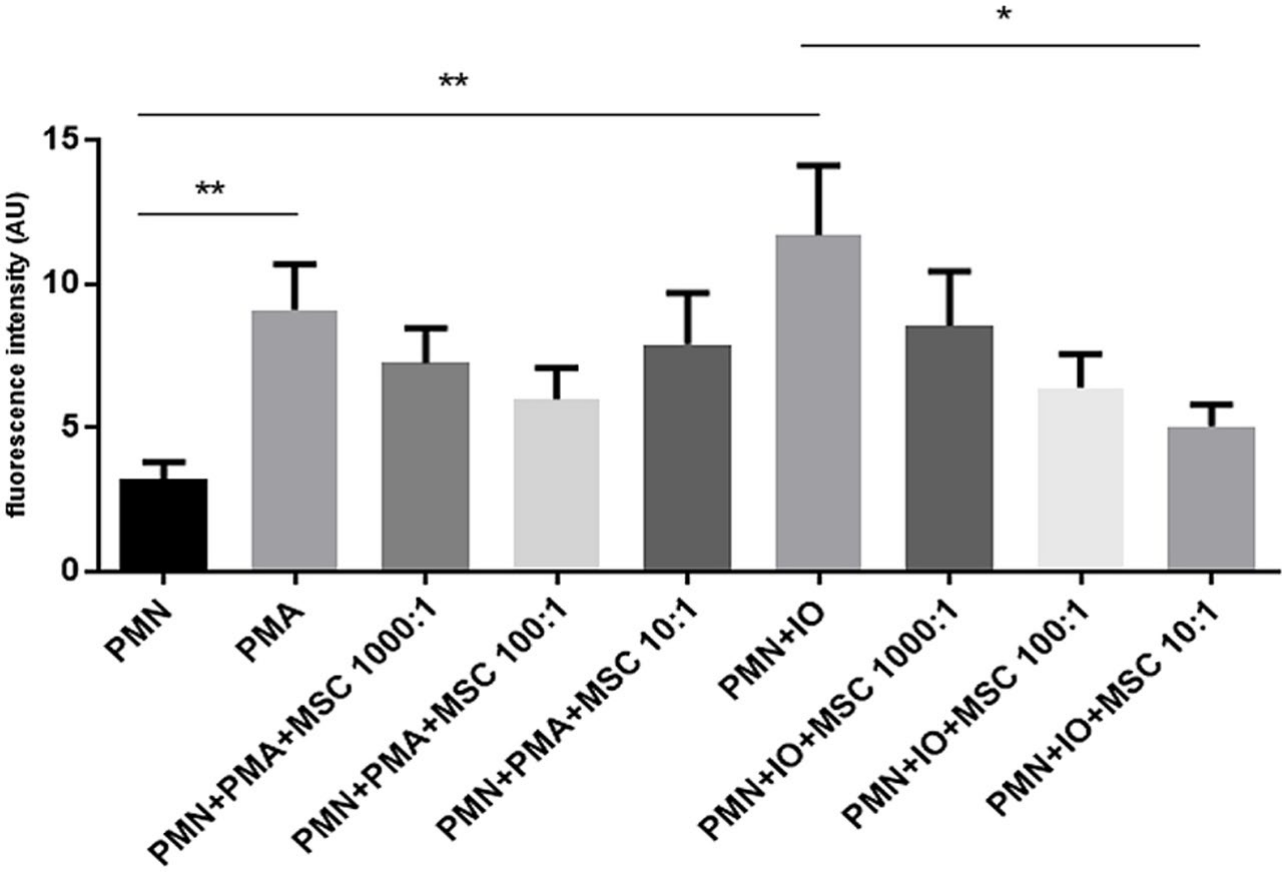
Inhibition of DNA release by equine polymorphonuclear neutrophils (PMN) is dependent on the adipose-derived stem cells (ADSCs) to PMN ratio. Bar graph showing fluorescence intensity of extracellular DNA, indicative of NETs released be equine PMN stimulated with PMA or ionomycin (Io) in the presence of ADSCs at different ratios. Fluorescence intensity was quantified following co-incubation with ADSCs at PMN: ADSCs ratios of 1,000:1, 100:1 and 10:1. A tendency toward reduced fluorescence signal was observed at the lowest PMN: ADSCs ratio (10:1), suggesting a dose-dependent inhibitory effect of ADSCs on NET release by equine PMN. Data represent as mean ± SD. *p ≤ 0.05; **p < 0.01. MSC, mesenchymal stem cell.

To evaluate whether the inhibition of equine NET release depends on the ratio of ADSCs to which PMN are exposed, an extracellular DNA-release quantification assay based on Sytox Orange- fluorescence intensities was performed. Equine PMN exposed to ADSCs at different ratios (1,000:1; 100:1; 10:1), ionomycin (Io), PMA or a combination of ADSCs and one of the previous stimuli stated was assessed (Figure 4). Extracellular DNA was measured after a 3 h incubation period under the different conditions, reflecting a late phase of NET formation. Fluorescence intensity analysis showed a significant increase of extracellular DNA release for the PMA-activated PMN (*p* < 0.05) and Io-activated PMN (*p* < 0.05), when compared to unstimulated equine PMN (Figure 4). Furthermore, a significant decrease (*p* < 0.05) in extracellular DNA was observed for the Io-activated PMN co-cultured in the presence ADSCs in a 10:1 ratio, when compared to the Io-activated PMN condition (Figure 4). No significant differences (*p* < 0.05) were observed for the other Io-activated PMN conditions, independently of the PMN-ADSCs ratios studied (1,000:1; 100:1) (Figure 4). Regarding PMA stimulated cells, no significant differences (*p* > 0.05) were achieved between PMA-activated PMN and the different conditions of PMN co-cultured in the presence of ADSCs in a 1,000:1, 100:1 and 10:1 ratio (Figure 4).

## 4 Discussion

In the present study, we evaluate the *in vitro* effects of equine ADSCs on equine PMN functions, specifically on ROS production and NET release. Our *in vitro* data demonstrated that co-culture of equine PMN with ADSCs significantly decreased NET release with no observable changes on ROS production. Furthermore, extracellular DNA quantification revealed that inhibition of NET release by equine PMN is dependent on the ADSCs to PMN ratio. Together, our findings support the capacity of ADSCs to influence equine NET formation and release.

NET formations were confirmed via SEM- and IFM analyses by the positive fluorescent co-localization of the anti-microbial proteins NE, histones and extracellular DNA. SEM analysis showed typical NET phenotype, i.e., chromatin fibers, not membrane bound and with granular aspect (38). IFM images and analysis further confirmed these findings, as ADSCs significantly decreased PMA-induced NET formation. Our results align with previous studies where MSCs have been shown to modulate PMN activation and NETosis in different species (31, 39, 40). In the mouse model, PMN in the presence of MSCs have been shown to significantly reduce NET release (31). Furthermore, a suppression of up to 63.5% in NETs releasing cells in PMA-activated human PMN when pre-treated with MSCs has been reported, with the higher suppression of NETs observed at 10:1 PMN-to-MSC ratio (31). Together, these results support the hypothesis that the modulatory and inhibitory effect of MSC upon NETs release may be considered as a cross-species conserved mechanism.

In mammalian PMN, ROS production via NADPH oxidase (NOX) is widely considered as an important triggering signal for the NETosis process (41). In the present study, we evaluate PMA-ROS production by equine PMN following exposure to ADSCs through a luminol-based chemiluminescence method. Contrary to reports in the literature describing an inhibitory role of MSCs on PMN oxidative burst (31, 42), our results showed that PMA-induced ROS production was not significantly alter after co-culture of equine PMN with ADSCs. These divergent results highlight the complexity of MSC–PMN interactions and emphasizes the need for further investigation. Previous studies have reported that MSCs suppress PMN-ROS production through the secretion of anti-inflammatory mediators such as prostaglandin E₂ (PGE₂), interleukin-10 (IL-10), and transforming growth factor-beta (TGF-*β*), which may impair and downregulate NOX activity (43, 44). This downregulation effect of MSC on PMN-derived ROS production has been described in different species including humans, mice, cats and horses (27, 31, 34, 45). However, our data did not show any significant changes in ROS levels following ADSCs co-culture, suggesting that in equine PMN, ADSCs-mediated immunomodulation may preferentially target other cellular functions rather than the oxidative burst. Different ROS production results may be attributed to different experimental settings and MSC source. Further studies are required to better understand and elucidate the exact pathways involved in ROS production inhibition within equine PMN. Interestingly, despite the lack of MSC-derived hampering effects on ROS production, our study showed that MSCs significantly reduce NET formation in co-cultured PMN, indicating that ADSCs-mediated regulation of PMN may occur independently of oxidative burst modulation.

NET formation and release by equine PMN in the presence of ADSCs was evaluated using two NET-inducing stimuli, PMA and Io. These stimuli activate PMN through two different signaling pathways, via protein kinase C (PKC) activation- a ROS dependent pathway for PMA and via calcium influx- a ROS independent pathway for Io. These stimuli allow us to evaluate and compare the modulatory effect of ADSCs on two different well-characterized NETosis-triggering pathways. Our results show a consistent tendency to decreased extracellular DNA released by PMN when co-cultured with ADSCs, with a maximum and significant reduction in NETs released for Io at a 10:1 ratio (PMN: ADSCs). For PMA, the same tendency is observed, but with a slight increase in extracellular DNA released by equine PMN when co-cultured with ADSCs at the lowest ratio (10:1). Consistent with previous studies demonstrating the immunomodulatory as well as anti-inflammatory properties of MSCs (42, 46), our findings provide additional evidence that ADSCs may influence PMN-NETosis. Interestingly, PMA-induced NETosis, which is known to be heavily dependent on NOX activation and ROS production (47), was not significantly inhibited by the presence of ADSCs for any of the three different ratios studied. These results were complementary and in accordance with our ROS results, when co-cultured with ADSCs did not downregulate ROS production in PMA-activated PMN. Contrary to our findings, other related studies in horses stated a decrease in ROS production linked to MSCs exposure, independently of the MSC source (34, 39, 48). Discrepancy between results may be attributed to different experimental settings, donor variability and origin of the MSCs tested, as other sources such as bone marrow and muscle-derived MSCs were studied. Nevertheless, our results highlight the need for further research to standardize the effect of MSCs on PMN effector mechanism within the equine system. On the other hand, Io-induced NET formation, was attenuated in a dose-dependent manner. This ROS independent pathway promotes the release of NET via calcium influx and PAD4 activation, leading to chromatin decondensation and histone release within the NETs. The potent microbicidal capacity of histones released within NET scaffolds is well known to induce endothelial cell damage (23, 49). Excessive and uncontrolled NET release have been implicated in the pathogenesis of multiple inflammatory, autoimmune, thrombotic and infectious diseases in humans (50, 51). In horses, this subject continues to be an emerging and active area of investigation, but dysregulated NET release has been linked to equine asthma, sepsis and laminitis among other diseases (52–54). Pharmacological inhibition of PAD4 downregulates chromatin decondensation and limits the extrusion of DNA into the extracellular space, resulting in reduced NET formation (55). Irrespective of the stimulation route, our results evidence the immunomodulatory effect of ADSCs upon the NETosis process suggesting a ADSCs-mediated inhibition of NET formation but dependent on the ADSCs: PMN ratio. Although different pathways are triggered with these two stimuli here used, i.e., Io and PMA, the observed reduction of equine NETs release, may suggest that ADSCs exert their effect in a common upstream signaling component for both pathways.

In summary, our results provide evidence of ADSCs-derived effects on equine PMN, particularly in their capacity to attenuate NET formation and release. These results support the potential role of ADSCs to interact with the innate immune response and maintaining immune homeostasis. ADSCs-derived effects such as attenuated NETs release may be significant for the development of future anti-inflammatory therapies in equine medicine. Further *in vitro*- and *ex vivo* studies are needed to characterize in more detail the effects of ADSCs on the different PMN-derived defence mechanisms (such as cytokine/chemokine secretion, purinergic- and MCT receptor expression) to understand and elucidate the specific molecular pathways involved in the modulation of equine NETosis.

## Data Availability

The raw data supporting the conclusions of this article will be made available by the authors, without undue reservation.
